# Inhibitors of CLK Protein Kinases Suppress Cell Growth and Induce Apoptosis by Modulating Pre-mRNA Splicing

**DOI:** 10.1371/journal.pone.0116929

**Published:** 2015-01-12

**Authors:** Shinsuke Araki, Ryo Dairiki, Yusuke Nakayama, Aiko Murai, Risa Miyashita, Misa Iwatani, Toshiyuki Nomura, Osamu Nakanishi

**Affiliations:** Pharmaceutical Research Division, Takeda Pharmaceutical Company Limited, Kanagawa, Japan; Georgia Regents University, UNITED STATES

## Abstract

Accumulating evidence has demonstrated the importance of alternative splicing in various physiological processes, including the development of different diseases. CDC-like kinases (CLKs) and serine-arginine protein kinases (SRPKs) are components of the splicing machinery that are crucial for exon selection. The discovery of small molecule inhibitors against these kinases is of significant value, not only to delineate the molecular mechanisms of splicing, but also to identify potential therapeutic opportunities. Here we describe a series of small molecules that inhibit CLKs and SRPKs and thereby modulate pre-mRNA splicing. Treatment with these small molecules (Cpd-1, Cpd-2, or Cpd-3) significantly reduced the levels of endogenous phosphorylated SR proteins and caused enlargement of nuclear speckles in MDA-MB-468 cells. Additionally, the compounds resulted in splicing alterations of *RPS6KB1* (*S6K*), and subsequent depletion of S6K protein. Interestingly, the activity of compounds selective for CLKs was well correlated with the activity for modulating *S6K* splicing as well as growth inhibition of cancer cells. A comprehensive mRNA sequencing approach revealed that the inhibitors induced splicing alterations and protein depletion for multiple genes, including those involved in growth and survival pathways such as S6K, EGFR, EIF3D, and PARP. Fluorescence pulse-chase labeling analyses demonstrated that isoforms with premature termination codons generated after treatment with the CLK inhibitors were degraded much faster than canonical mRNAs. Taken together, these results suggest that CLK inhibitors exhibit growth suppression and apoptosis induction through splicing alterations in genes involved in growth and survival. These small molecule inhibitors may be valuable tools for elucidating the molecular machinery of splicing and for the potential development of a novel class of antitumor agents.

## Introduction

Alternative pre-mRNA splicing is a critical molecular mechanism for generating diversity of the proteome and is essential in various biological processes such as differentiation, growth, and apoptosis [1, 2]. Pre-mRNA splicing is executed by the spliceosome, which comprises multicomponent ribonucleoprotein complexes (snRNPs) composed of five small nuclear RNAs (snRNAs) and a large number of associated proteins [3, 4]. The spliceosome is assembled and activated through a series of ATP/GTP-dependent steps from complex E to complexes A, B, and C by RNA-RNA and RNA-protein interactions. Additional auxiliary factors, Ser/Arg-rich (SR) proteins, play important roles in exon selection together with snRNPs. SR proteins such as SRSF1 and SRSF2 bind to an exonic splicing enhancer (ESE) or intronic splicing enhancer (ISE) to promote exon selection by recruiting spliceosomal components to the 3′ splice site of introns [3, 4]. SR proteins contain one or two RNA recognition motifs (RRMs) at their N-terminus and a single Arg/Ser-rich (RS) domain at their C-terminus [5, 6]. Members of kinase families including serine-arginine protein kinases (SRPKs) and CDC-like kinases (CLKs) phosphorylate the RS domains of SR proteins, thereby regulating their subcellular localizations and interactions with ESEs or ISEs of pre-mRNAs [7, 8].

Dysregulation of alternative splicing is frequently found in human diseases, including neurodegenerative diseases, autoimmune diseases, and tumors [9–11]. In cancer in particular, corroborative examples have demonstrated the relationships of splicing factors with disease progression. For instance, expression of SRSF1 is upregulated in colorectal cancer leading to tumor growth, and modulation of alternative splicing of *RPS6KB1* (*S6K)*, *BIN1*, and *MNK2* mRNAs is observed in breast cancers [12, 13]. Interestingly, *SRPK1* expression was reported to be upregulated in breast, pancreatic, and colorectal cancers [14–16]. Furthermore, whole-exome analyses of certain types of hematologic and solid malignancies identified mutually exclusive somatic mutations in genes encoding key components of the splicing machinery, such as SF3B1, SRSF2, and U2AF35 [17]. These findings suggest that abnormal function of the core splicing machinery can be a major driver of tumor pathogenesis.

Significant efforts have been made to identify splicing inhibitors using molecular targeting and chemical biology approaches. For instance, the CLK inhibitor TG003 was reported to modulate alternative splicing of SC35 (SRSF2) and CLK pre-mRNAs [18]. Additionally, novel natural products with different chemical structures, such as spliceostatin A (SSA), E7107, and herboxidiene, have been identified as anticancer agents, all of which directly target SF3B1 [19–21]. These compounds have been used as tools to dissect the function of the splicing machinery and in the development of novel anticancer agents. However, despite these efforts, little is known about the regulatory mechanisms of splicing-related kinases and their associations with diseases.

Here we report a series of small molecule inhibitors of the CLK family that induce large-scale splicing alterations in a number of transcripts, particularly those involved in growth and survival signaling. These compounds were found to inhibit cell growth and induce apoptosis in cancer cells by depleting their proteomes. Our data suggest that CLK family kinases play an essential role in the growth of cancer cells. These compounds will be valuable for understanding the molecular mechanism of splicing and may serve as potential seeds for the development of a novel class of cancer therapeutics.

## Results

### Identification of inhibitors of the kinase activities of CLK and SRPK family members

**Figure 1:**
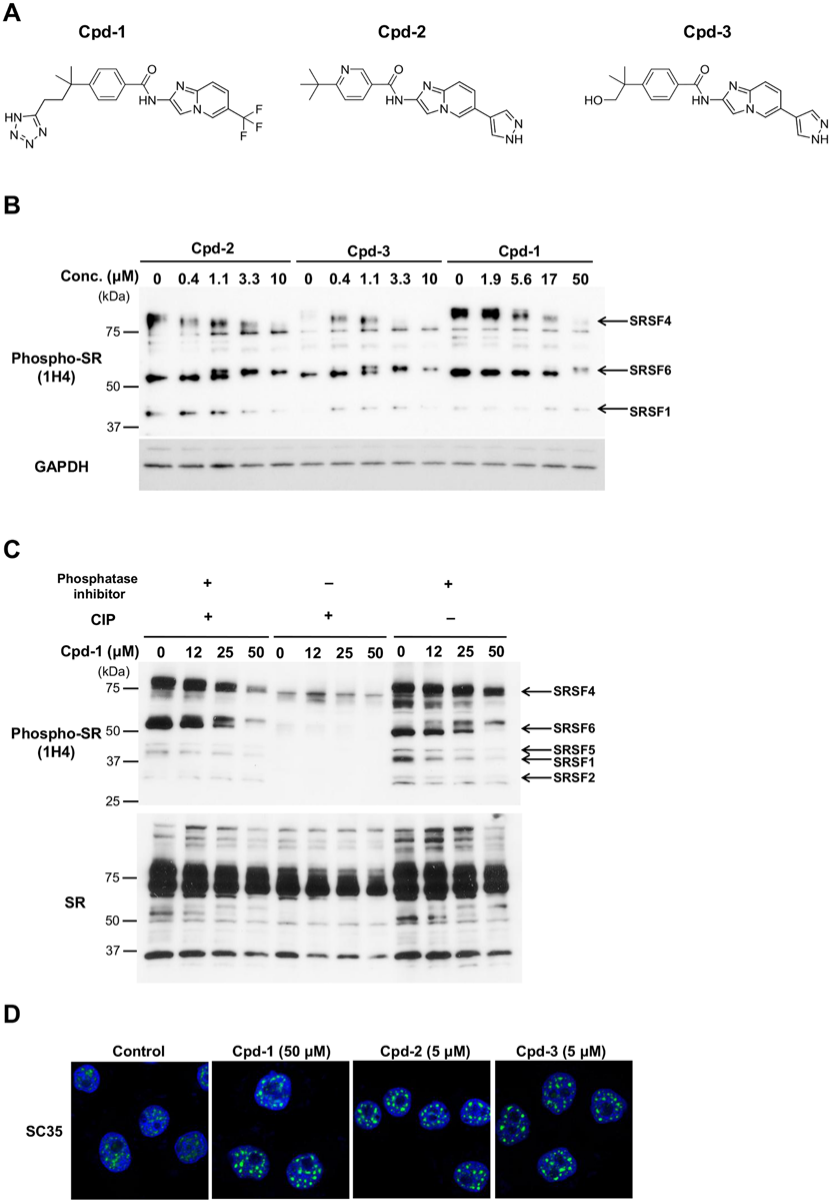
Cpd-1, Cpd-2, and Cpd-3 inhibit the phosphorylation of SR proteins. (A) Chemical structures of Cpd-1, Cpd-2, and Cpd-3. (B) MDA-MB-468 cells were treated with Cpd-1, Cpd-2, or Cpd-3 for 3 h. Immunoblot analyses were performed for phospho-SR isoforms (arrows). The positions of standard molecular weight markers are indicated on the left. The identity of each isoform was validated by analyzing cells transfected with the respective cognate SR siRNA (data not shown). (C) MDA-MB-468 cells were treated with Cpd-1 for 3 h. Cell lysates were treated with 3 U of CIP in the presence or absence of phosphatase inhibitors (PhosSTOP phosphatase inhibitor cocktail; Roche) for 1 h at 37°C. Immunoblot analyses were performed for SR and phospho-SR isoforms (arrows). (D) MDA-MB-468 cells were treated with Cpd-1, Cpd-2, or Cpd-3 for 6 h at the indicated concentrations. Nuclear speckles were visualized with an anti-SC35 antibody and detected using fluorescence microscopy. The data shown are representative of two to three independent experiments.

To identify inhibitors of splicing-related kinases, high-throughput screening was conducted with 870,000 compounds at a single concentration of 1 μM in luminescent *in vitro* kinase assays using SRPK1. A total of 319 active compounds satisfied the criteria to inhibit 30% of SRPK1 enzyme activity. We next evaluated inhibitory activity in *in vitro* kinase assays using CLK2 and identified three compounds, named as Compound-1 (Cpd-1), Compound-2 (Cpd-2), and Compound-3 (Cpd-3) (Fig. 1A and Table 1). Cpd-1 showed significant inhibitory activity against SRPK1 and SRPK2 with IC_50_ values of 61 and 75 nM, respectively (Table 1). Cpd-1 also possessed inhibitory activity against CLK1 and CLK2 with IC_50_ values of 16 and 45 nM, respectively (Table 1). Cpd-2 and Cpd-3 were also both highly potent and sensitive for CLK1 and CLK2, with 10-fold stronger activity than Cpd-1 (Table 1). Among the 28 critical oncology-related kinases, including CLK2, Cpd-1, Cpd-2 and Cpd-3 showed the strongest inhibitory activity against CLK2 (Table A in S1 File). Only three kinases were inhibited by more than 50% by 1 μM of the compounds. Cpd-1 and Cpd-3 moderately inhibited Aurora kinase A, and Cpd-2 moderately inhibited PI3Kα. These results suggest that the identified compounds have distinct inhibition profiles selective for SRPK and CLK.

**Table 1:** Specificity of the kinase inhibitors for CLK1, CLK2, SRPK1, SRPK2, and SRPK3.

**IC_50_ (nM)**	**Cpd-1**	**Cpd-2**	**Cpd-3**
CLK1	16	1.1	1.1
CLK2	45	2.4	2.1
SRPK1	61	200	130
SRPK2	75	310	260
SRPK3	10000	230	260

CLK1, SRPK1, SRPK2, and SRPK3 assays were performed with 5 µM ATP, and the CLK2 assay was performed with 20 µM ATP. The definition and determination of the IC_50_ values are described in the Methods.

To examine the cellular activity of these compounds, the phosphorylation levels of substrate SR proteins were analyzed using an anti-pan-phospho-SR antibody, which recognizes multiple classical members of the SR family [22]. All three compounds reduced the phosphorylation levels of SRSF4 (SRp75) in a concentration-dependent manner (Fig. 1B). Consistent with the differences in the IC_50_ values against CLKs, Cpd-2 and Cpd-3 inhibited phospho-SRSF4 at lower concentrations than Cpd-1. Treatment of cells with the three compounds also induced a significant mobility shift of SRSF6 (SRp55) from 55 kDa to approximately 60 kDa, consistent with previous findings [23, 24]. Phospho-SRSF1 (SF2) was decreased by Cpd-2 and Cpd-3, but not by Cpd-1, implying that CLK mainly controls the phosphorylation level of SRSF1 in MDA-MB-468 cells.

Treatment of cell lysates with alkaline phosphatase significantly suppressed the phosphorylation of all SR proteins, while treatment with a phosphatase inhibitor had little effect, confirming that the anti-phospho SR antibody selectively detected phosphorylated SR proteins (Fig. 1C). Notably, treatment with Cpd-1, Cpd-2, or Cpd-3 induced enlargement of nuclear speckles (Fig. 1D), which is characteristic of inhibition of the splicing machinery by expression of kinase-dead mutant forms of CLKs or SRPKs [23–26].

### Aberrant splicing of *S6K* mRNA in cells treated with Cpd-1

**Figure 2:**
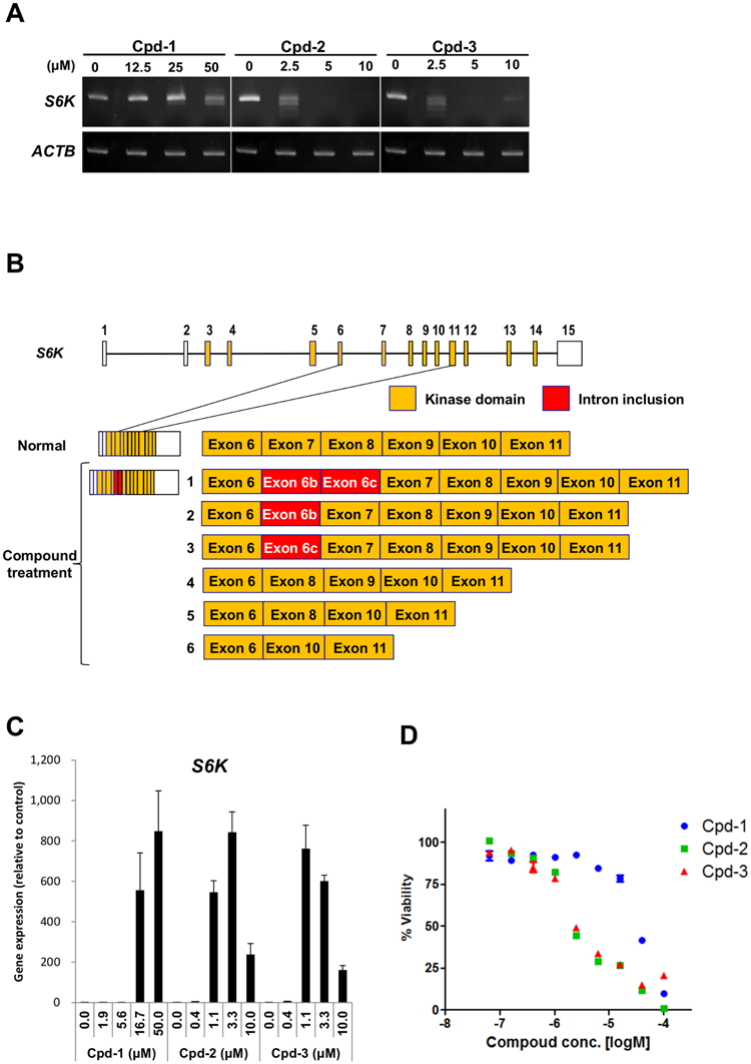
Alterations of *S6K* pre-mRNA splicing induced by Cpd-1, Cpd-2, and Cpd-3. (A) MDA-MB-468 cells were treated with Cpd-1, Cpd-2, or Cpd-3 for 72 h. Expression of *S6K* was detected by RT-PCR. *ACTB* mRNA expression was evaluated as an internal control. (B) Schematic representation of the alternatively spliced forms of *S6K* mRNAs upon treatment with the compounds. Each box represents an exon. Yellow boxes represent exons for the kinase domain of S6K and red boxes represent introns including a novel exon. (C) MDA-MB-468 cells were treated with Cpd-1, Cpd-2, or Cpd-3 for 6 h at the indicated concentrations. Quantitative RT-PCR analyses were performed for the expression of *S6K* mRNA exons 6–8 (exon 7 skipped). The data represent means ± SD from three independent analyses. (D) Cell viability after treatment with Cpd-1, Cpd-2, or Cpd-3. MDA-MB-468 cells were treated with each compound for 72 h at the indicated concentrations.

Next, we examined whether treatment of cells with the three compounds affected the splicing pattern of a target pre-mRNA. We selected *S6K* pre-mRNA, as knockdown of SRSF1, a downstream substrate of CLKs and SRPKs, was reported to alter the splicing of *S6K* [13]. RT-PCR analyses detected multiple mRNA bands when cells were treated with the three compounds (Fig. 2A). Each band was extracted and subjected to DNA sequencing. Results showed that the compounds induced the generation of six novel mRNA isoforms as well as the canonical form of *S6K* pre-mRNA (Fig. 2B). These alternatively spliced transcripts were classified into two types of splicing patterns: “intron-inclusion” and “exon-skipping”. There were three variants for the intron-inclusion forms (isoforms 1, 2, and 3) using cryptic splice sites located upstream of the exons. There were also three variants for the exon-skipping forms (isoforms 4, 5, and 6). Because these novel isoforms of *S6K* can be useful for distinguishing the potency of the compounds, we established quantitative RT-PCR analyses to measure the expression levels of the exon 7-skipped type of *S6K* pre-mRNA. Cpd-2 and Cpd-3, which were highly selective for CLK1/2, induced alternative splicing of the *S6K* pre-mRNA at lower concentrations than Cpd-1 (Fig. 2C). The expression level of the alternatively spliced *S6K* mRNAs was highest in cells treated with 1.1–3.3 μM Cpd-2 and Cpd-3. Interestingly, the expression level of the skipped transcripts correlated with the growth inhibitory activity (GI_50_ = 3.0 and 3.4 μM, respectively; Fig. 2D). The discovery of these novel splice variants of *S6K* pre-mRNA enabled us to characterize the activity of the three compounds. Notably, the mRNA isoforms expressed in cells treated with Cpd-1 had different sequences from those induced by knockdown of SRSF1 [13]. The function of SRSF1 is known to be regulated by phosphorylation by SRPKs and CLKs as well as methylation of arginine residues within the RRM domain [27]. It is of interest to clarify how methylation and phosphorylation contribute to the alternative splicing.

### Inhibition of CLK activity is responsible for inducing alternative splicing of *S6K*

**Figure 3:**
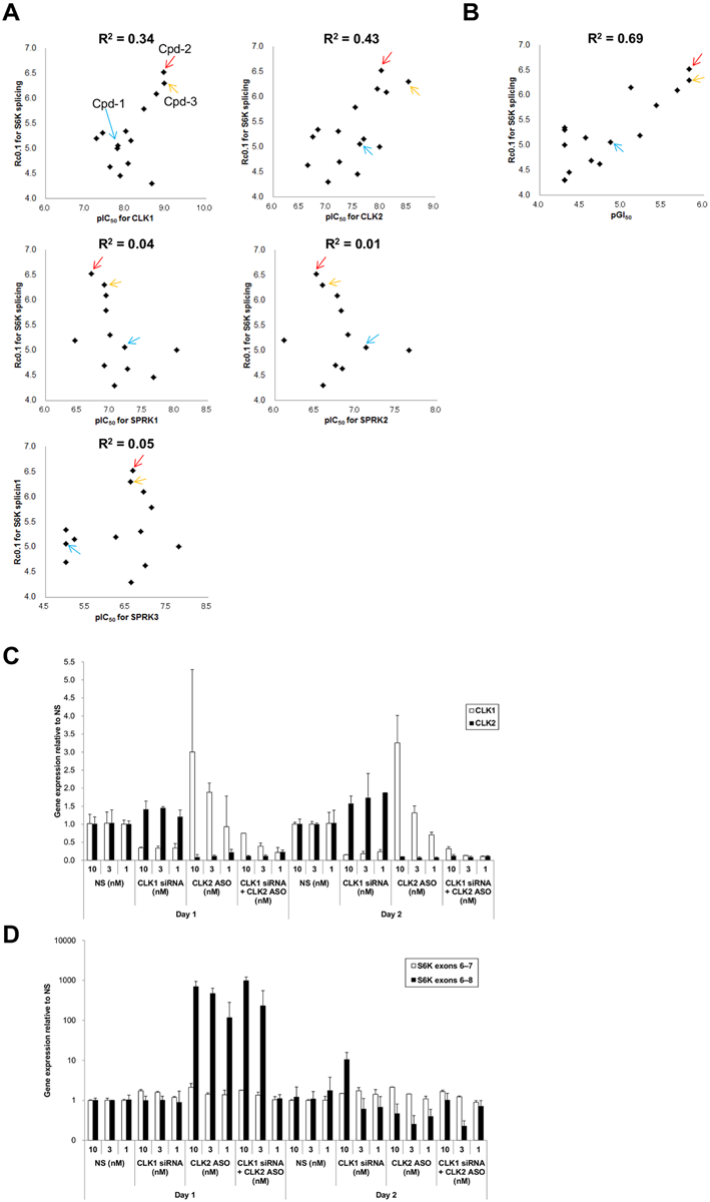
Correlation between splicing activity and cell growth by CLK1 and CLK2. (A) Scatter plots comparing *in vitro* kinase inhibition with cellular splicing alteration of *S6K* after treatment with Cpd-1, Cpd-2, Cpd-3, and a series of other compounds. The X-axis shows the pIC_50_ [−log_10_(IC_50_)] value for CLK1, CLK2, SRPK1, SRPK2, or SRPK3 inhibition in cell-free enzymatic assays. The Y-axis shows the splicing induction activity defined as the drug concentration (μM) that induced 10% of the copy number of the aberrantly spliced *S6K* mRNA without skipping exon 7 compared with the copy number of the canonical mRNA (Rc0.1). R^2^: coefficient of determination; blue arrow: Cpd-1; red arrow: Cpd-2; orange arrow: Cpd-3. (B) Scatter plot analysis comparing GI_50_ values (concentration required to inhibit growth by 50%) on the X-axis with Rc0.1 values on the Y-axis for the same compounds shown in (A). Blue arrow: Cpd-1; red arrow: Cpd-2; orange arrow: Cpd-3. (C, D) MDA-MB-468 cells were transfected with *CLK1* siRNA, *CLK2* antisense oligonucleotide (ASO), or control Non-Silencing siRNA (NS) at the indicated concentrations. Cells were harvested after 24 and 48 h. The data represent means ± SD from three independent experiments. (C) The expression levels of *CLK1* and *CLK2* were measured by quantitative RT-PCR. (D) ASO and siRNA transfection experiments were performed to identify the kinases that altered the splicing pattern of *S6K* pre-mRNA. RT-PCR analyses of the expression levels of *S6K* mRNA exons 6–7 (canonical mRNA) and exons 6–8 (aberrantly spliced mRNA lacking skipped exon 7).

To examine whether inhibition of a particular CLK and/or SRPK contributed to the alternative splicing of *S6K* pre-mRNA, we used an additional series of compounds with the same scaffold as Cpd-1, Cpd-2, and Cpd-3, but different selectivity. First, we examined the correlation between the enzymatic activity and the level of splicing alterations of *S6K* pre-mRNA for the individual compounds. We used the exon 7-skipped type of *S6K* variant to calibrate Rc0.1 values, i.e., the concentrations that induced 10% of the copy number of an alternative isoform compared with that of the canonical isoform. The inhibition of CLK1 and CLK2 activities was correlated with the splicing alteration activities (Rc0.1) (R^2^ = 0.3353 and 0.4255, respectively). In contrast, there was no correlation between the activities of SRPKs and the Rc0.1 values (R^2^ < 0.0468) (Fig. 3A). Next, we compared the levels of splicing alteration and growth inhibition of the compounds. The Rc0.1 values were significantly correlated with the GI_50_ values in MDA-MB-468 cells (R^2^ = 0.69; Fig. 3B). Similar correlations between Rc0.1 and GI_50_ values were also obtained in seven different cancer cell lines after treatment with Cpd-2 or Cpd-3 (Table 2). These results clearly suggest that inhibition of CLKs is the major driver for inducing alternative splicing of *S6K* pre-mRNA and subsequent inhibition of cell growth in a variety of cancer cell types.

**Table 2:** GI_50_ values and Rc0.1 values of the compounds for treatment of cancer cell lines.

**Cell name**	**Cell type**	**Cpd-1 (μM)**		**Cpd-2 (μM)**		**Cpd-3 (μM)**	
		**GI_50_**	**Rc0.1**	**GI_50_**	**Rc0.1**	**GI_50_**	**Rc0.1**
MDA-MB-468	breast cancer	30.5	8.6	3.0	0.3	3.4	0.5
A549	non-small cell lung cancer	102.3	>10	1.9	0.3	2.6	1.2
COLO205	colorectal cancer	27.3	>10	1.7	1.6	2.1	2.6
HCT-116	colorectal cancer	70.2	>10	2.2	1.2	2.5	1.9
NCI-H23	non-small cell lung cancer	32.0	>10	1.4	0.9	2.2	1.1
SW620	colorectal cancer	41.8	>10	2.0	1.2	2.9	1.4
COLO320DM	colorectal cancer	43.0	>10	0.6	0.9	1.5	1.1

The GI_50_ value represents the concentration required to achieve 50% growth inhibition.

The Rc0.1 value indicates that the ratio of the copy number of *S6K* exons 6–8 to that of *S6K* exons 6–7 is 0.1.

This was further confirmed using mRNA knockdown techniques. An antisense *CLK2* oligonucleotide, locked nucleic acid (LNA), was used, because the inhibitory activities of *CLK2* siRNAs were insufficient. First, we confirmed that the knockdown efficiencies for both *CLK1* and *CLK2* mRNAs were >80% (Fig. 3C). Depletion of *CLK1* or *CLK2* mRNAs, but not of those encoding SRPK family members, induced significant alterations in the splicing of *S6K* pre-mRNA on days 1 and 2 (Fig. 3D and Fig. A in S1 File). These data clearly suggest that inhibition of CLK1 and CLK2 plays a major role in altering the splicing pattern of *S6K* pre-mRNA and inhibiting cell growth.

### Inhibition of CLK activity alters global splicing patterns

**Figure 4:**
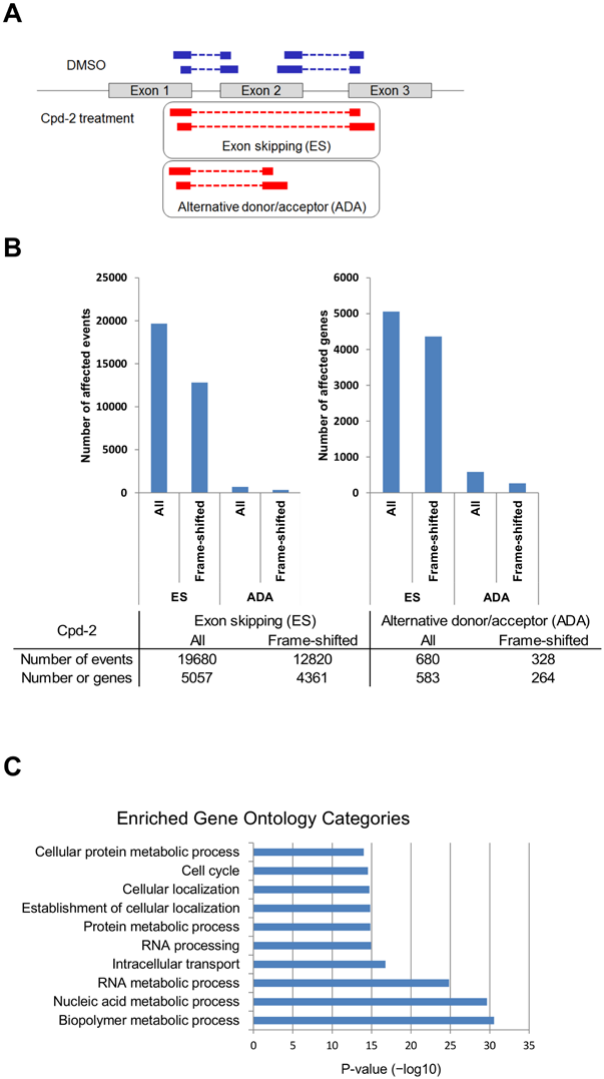
Induction of alternative splicing by CLK inhibitors. (A) Schematic representation of the alternative splicing induced by the CLK inhibitors. RNA-seq reads were aligned to exon junctions and classified as exon skipping (ES)-type splicing or alternative donor/acceptor (ADA)-type splicing. The junction sequences generated in cells treated with an inhibitor are shown as red boxes. (B) Numbers of genes and events with expression of alternatively spliced transcripts induced by Cpd-2 based on the RNA-seq data. MDA-MB-468 cells were treated with 5 μM Cpd-2 for 24 h. The genes or events with expression of ES-type splicing and ADA-type splicing were further classified into genes and events whose transcripts generated frameshifts. (C) Gene Ontology analyses of genes that expressed frameshifted transcripts induced by exon skipping after treatment with Cpd-1 for 24 h. The functional categories (Y-axis) and corresponding P-values (X-axis) are shown.

To identify alternatively spliced mRNAs affected by CLK inhibition, whole transcriptome analyses were performed after treatment with Cpd-2. The sequences of exon-exon junctions were aligned to 100 million paired-end reads and grouped into two classes: exon skipping (ES) and alternative donor/acceptor (ADA) (Fig. 4A). The genes with the largest number of reads of alternative junctions affected by Cpd-2 are summarized in Tables B and C in S1 File. Treatment with Cpd-2 for 24 h resulted in a large number of pre-mRNAs affected by alternative ES and ADA splicing events. ES events occurred 30 times more frequently than ADA events, and approximately 60% of the total ES splicing events generated frameshifts and premature termination codons (PTCs) downstream of the junctions (Fig. 4B).

To further examine whether a particular biological process is sensitive to the regulation of splicing, Gene Ontology analyses were performed for the genes whose splicing was affected by the CLK inhibitors, i.e., splice variants with exon skipping harboring PTCs. The results showed that genes in metabolic processes and cell cycle pathways were the most impacted, all of which play roles in growth and survival signaling (Fig. 4C and Table D in S1 File). The genes identified in pathways enriched by CLK inhibitor treatment are summarized in Table E in S1 File.

### Rapid degradation of aberrant mRNA isoforms harboring PTCs

**Figure 5:**
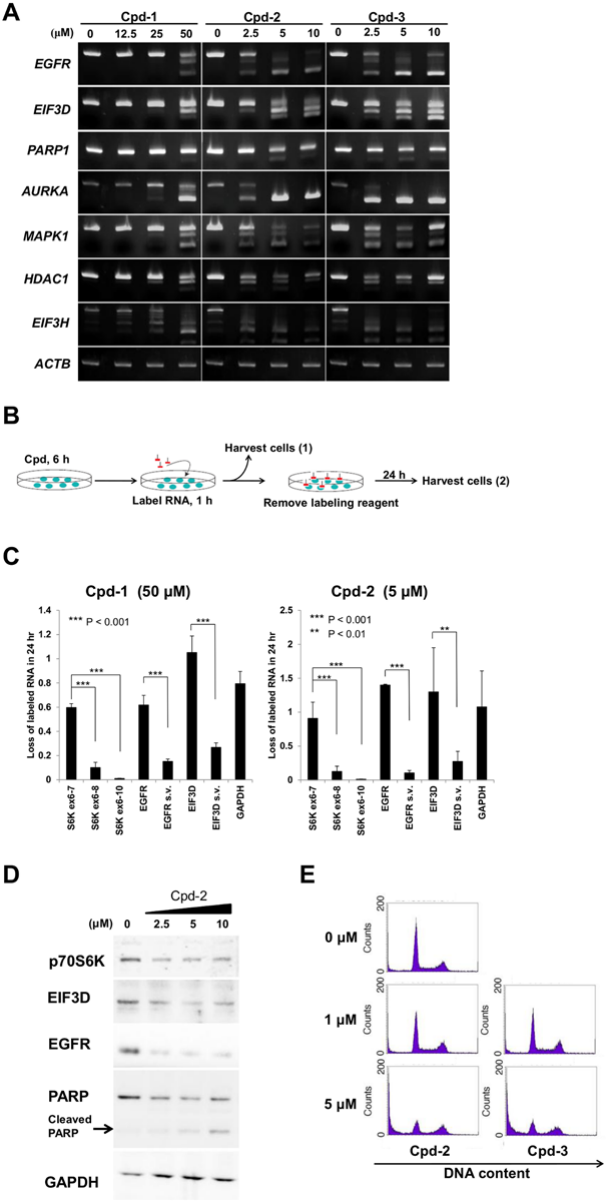
Rapid decay of mRNA splicing variants in cells treated with CLK inhibitors. (A) MDA-MB-468 cells were treated with Cpd-1, Cpd-2, or Cpd-3 for 24 h at the indicated concentrations. Total RNA was analyzed by RT-PCR. (B) Schematic representation of the RNA decay assay. MDA-MB-468 cells were treated with each compound for 6 h and cellular RNA was labeled for 1 h. After removal of the labeling reagents, the cells were cultured for a further 24 h. The cells were harvested before, harvest (1), and after, harvest (2), the chase and the labeled mRNA was purified. (C) Loss of labeled mRNAs of the indicated genes after the 24 h chase. The ratios of the amounts of the mRNAs at harvest (2) to those at harvest (1) were calculated for the individual splice isoforms using quantitative RT-PCR. The cells were treated with 50 μM Cpd-1 (left) or 5 μM Cpd-2 (right) before RNA labeling. The data represent means ± SD from three independent analyses. Statistical analyses were performed using an unpaired Student’s *t*-test (**P < 0.01; ***P < 0.001). s.v.: splicing variant. (D) Cpd-2 was added to MDA-MB-468 cells for 24 h at the indicated concentrations. Immunoblot analyses were performed. (E) MDA-MB-468 cells were treated with CLK inhibitors for 48 h. The cellular DNA contents were determined by flow cytometry. Representative data for three independent experiments are shown.

To more deeply understand the relevance of splicing modulation in biological responses, the alterations in the splicing pattern of key genes for growth and survival were examined by RT-PCR. Significant changes in the splicing of genes such as *EGFR*, *CD44*, *AURKA*, and *EIF3H* were observed (Fig. 5A). These spliced isoforms harboring PTCs were considered to stimulate nonsense-mediated mRNA decay (NMD) before translation. To determine whether aberrant mRNA isoforms harboring PTCs were degraded at a faster rate than the canonical transcripts, we performed pulse-chase labeling of cellular RNAs using 5-ethynyl uridine (EU) followed by quantitative RT-PCR to measure the amounts of mRNA isoforms (Fig. 5B). The amounts of *S6K* mRNA isoforms harboring PTCs, such as *S6K* exons 6–8 and *S6K* exons 6–10 induced by Cpd-1 or Cpd-2, were significantly lower than that of the canonical *S6K* exons 6–7, suggesting a higher turnover rate for mRNAs harboring PTCs (Fig. 5C). The *EGFR* and *EIF3D* mRNA isoforms with PTCs were also expressed at significantly lower levels than the canonical transcripts after treatment with Cpd-1 or Cpd-2. Consistent with the results for the rapid degradation of mRNAs, Cpd-2 treatment reduced the protein levels of S6K, EIF3D, and EGFR in a concentration-dependent manner, and no additional bands were identified, suggesting that none of the alternatively spliced mRNA isoforms induced by Cpd-2 were translated (Fig. 5D). It is conceivable that the degradation of PTC-containing mRNA isoforms is selectively accelerated by NMD and leads to depletion of the corresponding proteome required for cell growth and survival.

Interestingly, treatment with the CLK inhibitors led to the generation of cleaved PARP and alternative splicing of *PARP* pre-mRNA, resulting in the induction of apoptosis. Since the GO analyses highlighted an impact on the cell cycle (Fig. 4C), we further analyzed the cell cycle profiles upon treatment with the CLK inhibitors by flow cytometry. No cell cycle arrest was observed in treated MDA-MB-468 cells, while a significant increase in the sub-G1 fraction was confirmed (Fig. 5E). This effect on the cell cycle is inconsistent with previous reports that selective depletion of EGFR and S6K induces G1/S-specific cell cycle arrest [28, 29]. These results suggest that the splicing alterations and protein depletion of multiple factors for growth and survival contribute to the observed cellular effects of the CLK inhibitors in this cell line. Additionally, CLK inhibitors altered splicing patterns of *AURKA*, *HDAC1*, and *PARP*, all of which are critical factors of cell survival (Fig. 5A). The inhibitors for these molecules are reported to induce apoptosis in cancer cells [30–32], suggesting that apoptosis is induced by reduction of the expression of these apoptosis-related genes through splicing alteration.

## Discussion

To explore the molecular mechanisms of splicing-related kinases and seek potential therapeutic opportunities for cancer, we performed compound screening and identified a series of novel compounds that inhibit the kinase activities of CLK and SRPK family members. The isolation of these tool compounds enabled us to understand the roles of their biological effects in cell lines derived from human cancers. The compounds identified in this study induced large-scale splicing alterations that led to accelerated degradation of aberrantly spliced mRNAs harboring PTCs. This process resulted in depletion of the corresponding proteome essential to the survival of cancer cells, which in turn inhibited cell growth and induced apoptosis.

Comprehensive transcriptome analyses of cells treated with the kinase inhibitors showed that while the majority of these transcripts were spliced normally, the results identified alternatively spliced forms of transcripts of approximately 5000 genes, including those involved in growth and survival signaling, such as *S6K*, *EGFR*, *EIF3D*, *PARP1*, *AURKA*, *MAPK1*, and *EIF3H*. Notably, all the transcripts of *S6K* and *EIF3H* were decreased by higher concentrations of Cpd-2 and Cpd-3 as shown in Fig. 2A and Fig. 5A, while transcripts of *EGFR*, *PARP1*, and *AURKA* were still detected even at higher compound concentrations. These data argue against that possibility that the identified compounds have general transcription inhibition at higher concentrations. Although further study is required, it is conceivable that the alteration of splicing of the specific transcription factors that control transcription levels of *S6K* caused depletion of the overall transcription of *S6K* gene as a secondary effect of CLK inhibition. CLKs phosphorylate SR proteins and regulate alternative splicing by modulating the interactions of SR proteins with the ESEs and ISEs of pre-mRNAs via phosphorylation of RS domains within SR proteins [8]. Detailed comprehensive informatics analyses of the relationships between exon selection with primary amino acid sequences of the RS domains and the nucleotide sequences of ESEs/ISEs may reveal the basis of the gene-selective splicing alterations. Since CLKs have been suggested to target specific pre-mRNA sequences, it is of future interest to determine the precise molecular mechanisms responsible for generation of the aberrantly spliced transcripts.

We observed that inhibition of CLK activity primarily induced aberrant ES events that led to frameshifts. Conversely, inhibitors of SF3b complex E7107 and SSA were reported to induce intron inclusion by suppressing intron excision, which also led to a frameshift [19]. The SF3b complex comprises the U2 snRNP of complex A, and SSA arrests complex A immediately prior to activation, which is considered to terminate all reactions that occur downstream of complex A [33, 34]. In contrast, the inhibition of SR protein phosphorylation by the small molecules evaluated in this study may modulate the affinity of SR proteins for ESE/ISE sequences. Phospho-SR proteins lead to alternative exon selection upon activation of complex E before complex A is formed, but do not inhibit all of the downstream reactions. Therefore, the spectrum of the transcriptome modulated by the CLK inhibitors is expected to be different from that after SSA treatment. Direct comparisons between inhibition of CLKs and SF3b may elucidate the detailed mechanisms of the splicing reactions and help us to understand whether small molecule kinase inhibitors will be suitable for clinical applications.

One of the important findings of our study was the ability of the small molecule inhibitors to more efficiently inhibit the activities of CLK1/CLK2 compared with those of SRPK1/SRPK2, which was significantly correlated with the splicing alterations of *S6K* pre-mRNA as well as the inhibition of cancer cell growth. These results suggest that CLKs and SRPKs function differently in the splicing machinery through their substrate specificities, particularly for SR proteins. Moreover, CLKs may play a major role in ensuring the accurate splicing of pre-mRNAs that encode proteins involved in cell survival, such as S6K. In contrast, SRPK1 modulates alternative splicing of *VEGF* pre-mRNA, which leads to excessive angiogenesis in patients with Denys-Drash Syndrome harboring a mutation of the tumor suppressor gene *WT1* [35]. The effects of inhibition of SRPK1 and SRPK2 by the compounds may be revealed using *in vivo* tumor models with *WT1* mutations rather than cell culture models.

In conclusion, the newly discovered CLK inhibitors revealed high potency for growth inhibition and apoptosis induction in cells by modulating large-scale aberrant alternative splicing. These small molecule inhibitors are valuable tools for better understanding the molecular mechanisms of splicing. Furthermore, these inhibitors may serve as seeds for a novel class of cancer therapeutics. Future studies should also investigate the therapeutic potential of CLK inhibitors beyond the field of cancer treatment. CLK family kinases are also involved in alternative splicing and RNA processing in Duchenne muscular dystrophy, Alzheimer’s disease, HIV-1, and influenza virus [18, 36–38]. TG003 selectively inhibits CLK1 and CLK4, while our compounds (Cpd-2 and Cpd-3) selectively inhibit CLK1 and CLK2. TG003 is known to increase the production of the dystrophin protein without severe cytotoxicity, whereas our compounds show growth inhibition in cancer cells. How each inhibitor differentially impacts cellular functions will be the subject of further investigation. The availability of inhibitors with different selectivity will enable researchers to better understand the differences between CLK subclasses and to identify appropriate therapeutic opportunities.

## Materials and Methods

### Chemical synthesis

A series of compounds, Cpd-1, Cpd-2, and Cpd-3, were synthesized and purified according to the method described in a previous patent application [39]. Cpd-1, Cpd-2, and Cpd-3 correspond to examples 446, 114, and 464, respectively. Specifically, Cpd-1 is 4-(2-methyl-4-(1H-tetrazol-5-yl)butan-2-yl)-N-(6-(trifluoromethyl)imidazo[1,2-a]pyridin-2-yl)benzamide, Cpd-2 is 6-tert-butyl-N-(6-(1H-pyrazol-4-yl)imidazo[1,2-a]pyridin-2-yl)nicotinamide, and Cpd-3 is 4-(1-hydroxy-2-methylpropan-2-yl)-N-(6-(1H-pyrazol-4-yl)imidazo[1,2-a]pyridin-2-yl)benzamide.

### Preparation of enzymes

Full-length human recombinant SRPK1 with a FLAG tag linked to the N-terminus (FLAG-hSRPK1) was prepared using a baculovirus expression system. The recombinant baculovirus used to express FLAG-hSRPK1 was constructed according to the protocol provided with the Bac-to-Bac Baculovirus Expression System (Invitrogen, Carlsbad, CA). Sf9 cells were infected with the amplified recombinant baculovirus, cultured at 27°C for 3 days, harvested, and stored at −80°C. The frozen cells were suspended in lysis buffer [50 mM Tris-HCl pH 7.5, 150 mM NaCl, 10% (w/v) glycerol, 1 mM DTT, and complete protease inhibitor cocktail (Roche, Basel, Switzerland)], sonicated for 1 min, and centrifuged at 15,000 × *g* for 30 min. The soluble fraction was applied to an anti-FLAG M2 affinity gel (Sigma-Aldrich, St. Louis, MO), and FLAG-hSRPK1 was eluted with elution buffer [50 mM Tris-HCl pH 7.5, 10% (w/v) glycerol, 1 mM DTT, and 0.1 mg/mL FLAG peptide]. FLAG-hSRPK1 was further purified by anion exchange chromatography (MonoQ 5/50 GL; GE Healthcare, Piscataway, NJ) and gel filtration chromatography (HiLoad 26/60 Superdex 200 pg; GE Healthcare). Protein concentration was measured using BCA protein assay reagents (Thermo Scientific, Pittsburgh, PA). CLK1 and CLK2 were purchased from Life Technologies (Carlsbad, CA) and SRPK2 and SRPK3 were obtained from Millipore (Billerica, MA).

### *In vitro* kinase assays

The ATP concentration for each assay was adjusted to the K_m_ for each kinase. Kinase assays for SRPK1–3 and CLK1 were performed using the ADP-Glo™ assay (Promega, Madison, WI). The kinase buffer contained 25 mM HEPES (pH 7.5), 10 mM magnesium acetate, 1 mM DTT, 0.01% BSA, and 0.01% Tween-20. The reaction mixtures contained 50 ng/mL SRPK1, 100 ng/mL SRPK2 or SRPK3, or 300 ng/mL CLK1 and 3 μM substrate peptide [GRSRSRSRSRSRSRSR] (Thermo Scientific). After incubating the enzyme, peptide, and compounds for 10 min, kinase reactions were initiated by adding ATP to a final concentration of 5 µM, followed by incubation for 30 min (15 min for SRPK1). The kinase reactions were terminated by adding ADP-Glo™ reagent (Promega). ADP was detected using a kinase detection reagent (Promega). The kinase activity of CLK2 was measured using the LANCE® Ultra assay (Perkin-Elmer, Waltham, MA) with ADP-Glo assay buffer. The reaction mixtures contained 100 ng/mL CLK2 and 50 nM ULight™-myelin basic protein (MBP) peptide (Perkin-Elmer). After incubating the enzyme, peptide, and compounds for 5 min, kinase reactions were initiated by adding ATP to a final concentration of 20 μM, followed by incubation for 30 min. The enzyme reactions were terminated by adding 30 mM EDTA, and phosphorylated ULight-MBP peptides were detected using a europium-labeled anti-phospho-MBP antibody diluted with detection buffer (Perkin-Elmer). The plates were read using an EnVision 2102 multilabel reader (Perkin-Elmer). We defined the absorbance of the reaction without enzyme as 100% inhibitory activity and that of the complete reaction mixture as 0% inhibitory activity. The IC_50_ values were estimated using the “Sigmoidal dose-response (variable slope)” equation in GraphPad Prism 5 software (GraphPad Software, San Diego, CA), with the maximum and minimum of the curve constrained to 100 and 0, respectively.

### Kinase panel assay

Kinase panel assays were performed using the FRET-based Z’- LYTE enzymatic kinase assay system (Life Technologies, Carlsbad, CA), which was carried out using the SelectScreen Kinase Profiling Service (Life Technologies). The standard reaction contained 2 μM peptide substrate, 100 μM ATP, 50 mM HEPES (pH 7.5), 0.01% Brij-35, 10 mM MgCl_2_, 1 mM EGTA, and each kinase. Cpd-1, Cpd-2, and Cpd-3 at two point concentration (100 and 1000 nM) with duplicate data points (n = 2) were assayed following the detailed procedures outlined in the SelectScreen. The full assay protocol and conditions can be obtained at www.lifetechnologies.com. Percent inhibition represents a ratio of phosphorylated product formed in the presence of the compound compared with phosphorylated product formed in reactions containing 1% DMSO. Inhibition data indicates the mean value of kinase inhibition from duplicate data.

### Cell culture

The human colorectal cancer cell line HCT116 and human lung cancer cell line A549 were maintained in Dulbecco’s Modified Eagle Medium (Invitrogen). The human colorectal cancer cell lines COLO205 and COLO320DM and human lung cancer cell line NCI-H23 were maintained in RPMI 1640 (Invitrogen). The human breast cancer cell line MDA-MB-468 was maintained in Leibovitz’s L-15 medium (Invitrogen). The media were supplemented with 10% FBS (JRH), penicillin (10,000 U/mL; Invitrogen), and streptomycin (10,000 μg/mL; Invitrogen). The cells were seeded and subcultured in 100-mm diameter dishes every 3–4 days. All cells were purchased from American Type Culture Collection (Manassas, VA).

### Immunofluorescence analysis

MDA-MB-468 cells were fixed with 4% paraformaldehyde for 30 min at room temperature, washed with PBS, blocked with 5% BSA in PBS, incubated with an anti-SC35 antibody (Abcam, Cambridge, UK) for 1 h at room temperature, and incubated with a secondary antibody for 1 h. The cells were mounted and counterstained with DAPI (Vector Laboratories, Burlingame, CA) and detected using fluorescence microscopy.

### RT-PCR

Total RNA was extracted using an RNeasy Miniprep Kit (Qiagen, Valencia, CA) in accordance with the manufacturer’s instructions. Reverse transcription was performed using a TaqMan Reverse Transcription Reagents Kit (Applied Biosystems, Carlsbad, CA). The PCR conditions were as follows: one cycle of 5 min at 95°C; 35 cycles of 30 s at 94°C, 30 s at 60°C, and 30 s at 72°C; and one cycle of 10 min at 72°C. The PCR products were separated in 2% agarose gels. Real-time PCR was performed using a TaqMan MGB probe (Applied Biosystems), and the amount of FAM fluorescence was measured as a function of the PCR cycle number (C_T_) using an ABI 7900 Real-time PCR System (Applied Biosystems). The expression level of each mRNA was normalized by that of *GAPDH* mRNA. All primer and probe sequences are listed in Table F in S1 File.

### Cell viability assay

MDA-MB-468 cells (5 × 10^3^) were seeded into 96-well plates and incubated overnight in 50 μL of culture medium. Next, 50 µL of each compound diluted in culture medium was added to the wells. After 72 h of incubation, viability was determined using the CellTiter-Glo Luminescent Cell Viability Assay (Promega) according to the manufacturer’s instructions. The GI_50_ values were calculated using GraphPad Prism 5 software with a sigmoid dose-response curve.

### mRNA sequencing

Total RNA was extracted using an RNeasy Miniprep Kit (Qiagen), and the quality was ascertained by the presence of two distinct peaks at 18S and 28S with no additional peaks using a Bioanalyzer (Agilent Technology, Santa Clara, CA). The cells were treated with 50 μM Cpd-1 for 6, 24, or 72 h or with 5 μM Cpd-2 for 24 h. Illumina mRNA-Seq reads (75 nucleotides, 100 million reads) were analyzed using TopHat [40]. Each sequence was first aligned to GRCh37/hg19 in the human genome using Bowtie [41], allowing as many as two mismatches/indels (insertions or deletions). Reads that mapped to >40 loci were deleted, and the remaining reads were aligned to exon junctions. The aligned junction reads were classified into ES-type splicing and ADA-type splicing. Detection of frameshifts was performed as follows. First, ES-type splicing and ADA-type splicing events were selected, and the lengths of the middle exons (skipped or inserted) were analyzed *in silico*. Exons with 3n-2 and 3n-1 base pair lengths were assumed to cause frameshifts, and the splicing events with such middle exons were recorded as frameshift-producing splicing patterns (N represents any positive integer).

### Fluorescence pulse-chase RNA labeling assay

MDA-MB-468 cells (5 × 10^5^) were seeded into 6-well plates and incubated overnight. The cells were treated with each compound for 6 h and incubated with culture medium containing 5-EU (Invitrogen) to label cellular RNA. After incubation for 1 h, the medium was replaced with EU-free fresh medium and the cells were cultured for a further 24 h to allow degradation of the labeled RNA (chase). Cells were harvested before and after the chase and EU-labeled RNA was isolated using a Click-iT Nascent RNA Capture Kit (Invitrogen) according to the manufacturer’s instructions. The isolated RNA was analyzed by quantitative RT-PCR to measure the amount of each mRNA splice isoform.

### Transfection of cells with siRNAs and LNA

MDA-MB-468 cells were seeded into 96-well plates at a density of 4 × 10^3^ cells/well and incubated overnight in Leibovitz’s L-15 medium containing 10% FBS. *CLK1* siRNA, *SRPK1* siRNA, *SRPK2* siRNA, (ON TARGETplus SMARTpool siRNA; Dharmacon, Boulder, CO), *CLK2* LNA, or control Non-Silencing siRNA (ON TARGETplus SMARTpool siRNA; Dharmacon) was mixed with DharmaFECT 1 transfection reagent. The cells were transfected with each mixture for 72 h according to the manufacturer’s instructions, and cell lysates were prepared for real-time PCR.

### Immunoblot analysis

Cells were lysed in radioimmunoprecipitation assay buffer containing 50 mM Tris-HCl (pH 7.6), 150 mM NaCl, 1% Triton X-100, and protease inhibitor cocktail tablets (Roche). After removal of the insoluble matter by centrifugation at 15,000 rpm for 15 min, the protein concentrations of the supernatants were determined using a BCA Protein Assay Kit (Pierce Biotechnology, Rockford, IL). The lysates were directly combined with one-quarter volume of 5× sample buffer [25 mmol/L Tris-HCl pH 6.8, 10% glycerol, 0.8% SDS, and 0.004% bromophenol blue] and boiled at 100°C for 5 min. The samples were subjected to 5–20% gradient SDS-polyacrylamide gel electrophoresis, and the separated proteins were electrophoretically transferred to polyvinylidene difluoride membranes using an I-blot System (Invitrogen). The membranes were incubated with primary antibodies and visualized using enhanced chemiluminescence reagents (GE Healthcare). The primary antibodies were as follows: anti-phospho-SR (1H4), anti-SR (Invitrogen), anti-EGFR, anti-EIF3D, anti-GAPDH (Santa Cruz Biotechnology, Santa Cruz, CA), anti-S6K and anti-PARP (Cell Signaling Technology, Danvers, MA).

### Cell cycle analysis

MDA-MB-468 cells were cultured for specified periods of time, harvested, washed twice with ice-cold PBS, and treated with Cycle Test Plus DNA Reagents (BD Biosciences, Franklin Lakes, NJ) in accordance with the manufacturer’s instructions. The DNA contents were determined using a FACScan with CellQuest software (BD Biosciences).

## Supporting Information

S1 File Table A, Selectivity profile of Cpd-1, Cpd-2, and Cpd-3 for critical oncology-related kinases. **Table B**, Top 50 transcripts lacking exons through exon skipping in cells treated with Cpd-2 for 24 h. **Table C**, Top 50 transcripts with alternative donor/acceptor splice sites in cells treated with Cpd-2 for 24 h. **Table D**, Top 50 Gene Ontology results for genes lacking exons through exon skipping in cells treated with Cpd-2 for 24 h. The P-values were obtained by Fisher’s exact test for statistically significant differences. **Table E**, Top 50 transcripts, encoding proteins involved in cell growth and survival, lacking exons through exon skipping in cells treated with Cpd-2 for 24 h. **Table F**, List of primers, TaqMan MGB probes, and ASO sequences. **Fig. A**, Analysis of gene expression in cells treated with SRPK1 and SRPK2 siRNAs. MDA-MB-468 cells were transfected with *SRPK1* siRNA, *SRPK2* siRNA, or control Non-Silencing siRNA (NS). Cells were harvested after 24, 48, and 72 h of treatment. (A) The expression level of each gene was measured by quantitative RT-PCR. (B) The expression levels of *S6K* exons 6–7 (normal mRNA) and *S6K* exons 6–8 (aberrant splicing variant with skipped exon 7) were determined by quantitative RT-PCR. The data represent means ± SD from three independent analyses.
(DOC)
