# Supplementary material for: Inhibitors of CLK Protein Kinases Suppress Cell Growth and Induce Apoptosis by Modulating Pre-mRNA Splicing
Source: PLoS One. 2015 Jan 12;10(1):e0116929. doi: 10.1371/journal.pone.0116929 (PMC4291223; doi:10.1371/journal.pone.0116929)
Supplement: S1 File — Table B, Top 50 transcripts lacking exons through exon skipping in cells treated with Cpd-2 for 24 h. Table C, Top 50 transcripts with alternative donor/acceptor splice sites in cells treated with Cpd-2 for 24 h. Table D, Top 50 Gene Ontology results for genes lacking exons through exon skipping in cells treated with Cpd-2 for 24 h. The P-values were obtained by Fisher’s exact test for statistically significant differences. Table E, Top 50 transcripts, encoding proteins involved in cell growth and survival, lacking exons through exon skipping in cells treated with Cpd-2 for 24 h. Table F, List of primers, TaqMan MGB probes, and ASO sequences. Fig. A, Analysis of gene expression in cells treated with SRPK1 and SRPK2 siRNAs. MDA-MB-468 cells were transfected with SRPK1 siRNA, SRPK2 siRNA, or control Non-Silencing siRNA (NS). Cells were harvested after 24, 48, and 72 h of treatment. (A) The expression level of each gene was measured by quantitative RT-PCR. (B) The expression levels of S6K exons 6–7 (normal mRNA) and S6K exons 6–8 (aberrant splicing variant with skipped exon 7) were determined by quantitative RT-PCR. The data represent means ± SD from three independent analyses. (DOC) [file pone.0116929.s001.doc]

[Supplemental Data]

Inhibitors of CLK protein kinases suppress cell growth and induce apoptosis by modulating pre-mRNA splicing

**Shinsuke Araki*, Ryo Dairiki, Yusuke Nakayama, Aiko Murai, Risa Miyashita, Misa Iwatani, Toshiyuki Nomura, and Osamu Nakanishi^‡^**

Pharmaceutical Research Division, Takeda Pharmaceutical Company Limited, Kanagawa, Japan

^‡^Present address: National Institute of Biomedical Innovation, Osaka, Japan

*Corresponding author: Shinsuke Araki, Pharmaceutical Research Division, Takeda Pharmaceutical Company Limited, 26-1, Muraoka-Higashi 2-chome, Fujisawa, Kanagawa 251-8555, Japan

Tel: +81-466-32-2600; Fax: +81-466-29-4415; E-mail: shinsuke.araki@takeda.com

**Table A.** **Selectivity profile of Cpd-1, Cpd-2, and Cpd-3 for critical oncology-related kinases**

**Table B.** **Top 50 transcripts lacking exons through exon skipping in cells treated with Cpd-2 for 24 h**

**Table C.** **Top 50 transcripts with alternative donor/acceptor splice sites in cells treated with Cpd-2 for 24 h**

**Table D.** **Top 50 Gene Ontology results for** **genes lacking exons through exon skipping in cells treated with Cpd-2 for 24 h.** The P-values were obtained by Fisher’s exact test for statistically significant differences.

**Table E.** **Top 50 transcripts, encoding proteins involved in cell growth and survival, lacking exons through exon skipping in cells treated with Cpd-2 for 24 h**

**Table F.** **List of primers, TaqMan MGB probes, and ASO sequences**

RT-PCR primers (all 5′ to 3′)

S6K-F: GTGGAGGAGAACTATTTATGCAGTTAGAAAGAG

S6K-R: TCCAGCGTCCCCAGGACCAGCTC

EGFR-F: AAAAGTGTGATCCAAGCTGTCCCAAT

EGFR-R: GGGACACTTCTTCACGCAGGTG

EIF3D-F: GATGAGCTGTACCCGCTCAGTGTATTC

EIF3D-R: TGTCTTGATGTTGATGAAGGACACTTCC

PARP1-F: GGATGACCAGCAGAAAGTCAAGAAGAC

PARP1-R: TTATCCTCTGTAGCAAGGAGGCTGAAG

AURKA-F: GTCCAAAACGTGTTCTCGTGACTCAG

AURKA-R: AGGTCTCTTGGTATGTGTTTGCCTCAA

MAPK1-F: ATGATCACACAGGGTTCCTGACAGAAT

MAPK1-R: GCCAGAGCCTGTTCTACTTCAATCCTC

HDAC1-F: GTATCACCAGAGGGTGCTGTACATTGA

HDAC1-R: CCAGCATCAGCATAGGCAGGTTAAAG

EIF3H-F: ATTCAGCCGTGAAGCAAGTGCAGATAG

EIF3H-R: GTTTGAAGAGTTTGGACAGGTCCTCCT

ACTB-F: CCAGCTCACCATGGATGATGATATCG

ACTB-R: GGAGTTGAAGGTAGTTTCGTGGATGC

Quantitative RT-PCR primers and probes (all 5′ to 3′)

S6K ex6-7 F: TTATGCAGTTAGAAAGAGAGGGAATATTT

S6K ex6-7 R: CCCCAAAGCCATGGAGATTT

S6K ex6-7 probe: TGGAAGACACTGCCTGCTTTTACTTGG

S6K ex6a-8 F: TTATGCAGTTAGAAAGAGAGGGAATATTT

S6K ex6a-8 R: GATTCTTTGCATAGTCCAAAGTCTGT

S6K ex6a-8 probe: TGGAAGACACTGCCTGGTCATGTGAAA

S6K ex6a-10 F: GAAAGAGAGGGAATATTTATGGAAGACA

S6K ex6a-10 R: GGCAAATTGAGTTTACATTTGAGGAT

S6K ex6a-10 probe: TGCCTGCCCCCATTCACTGGG

EGFR F: CATACGCGGCAGGACCAA

EGFR R: TCAGGCTGACGACTGCAAGA

EGFR probe: CAACATGGTCAGTTTT

EIF3D F: GGGATATTGTCGTCCAGAGAGTTG

EIF3D R: GGGCTCATTGGCAGTCTCA

EIF3D probe: TTTGACCTCCTGACAGTGA

EGFR s.v. R: GCCGGGCAGGTCTTGAC

EGFR s.v. probe: AGCAACAGGACCAGACAA

EIF3D s.v. F: TGGGATATTGTCGTCCAGAGAGT

EIF3D s.v. R: CATGTCGTCCTCCACAAACG

EIF3D s.v. probe: ACTTTGGGGAAGGAAAGA

GAPDH F: GCACCACCAACTGCTTAGCA

GAPDH R: ACTGTGGTCATGAGTCCTTCCA

GAPDH probe: TCATCCATGACAACTTT

LNA sequence

CLK2 LNA sequence: CGAaatgctgctcCAC [n = DNA; N = LNA]

**A**

**B**
